# Pescadillo ribosomal biogenesis factor 1 reduction suppresses tumour growth and renders chemosensitivity of head and neck squamous cell carcinoma

**DOI:** 10.1002/cam4.5315

**Published:** 2022-10-11

**Authors:** Dapeng Li, Changyu Yao, Zhao Ding, Ping Liu, Xue Chen, Weiwei Liu, Fangzheng Yi, Chuanya Jiang, Hongwu Li, Yehai Liu, Jing Wu

**Affiliations:** ^1^ Department of Otolaryngology‐Head & Neck Surgery The First Affiliated Hospital of Anhui Medical University Hefei People's Republic of China; ^2^ Anhui Medical University Hefei People's Republic of China; ^3^ Graduate School of Anhui Medical University Hefei People's Republic of China; ^4^ Anhui Public Health Clinical Center Hefei People's Republic of China

**Keywords:** chemosensitivity, c‐Myc, PES1, proliferation

## Abstract

**Background:**

As one of the most devastating cancers, head and neck squamous cell carcinoma (HNSCC) has a short survival time and poor prognosis. Pescadillo ribosomal biogenesis factor 1 (PES1) plays a critical role in the progression of numerous cancers. However, its role and underlying mechanisms in HNSCC remain unclear.

**Methods:**

A variety of bioinformatic approaches were used to identify the expressions, prognostic and diagnostic value of PES1 in HNSCC. qRT‐PCR, immunofluorescence (IF) assay, western blotting and immunohistochemical (IHC) were used to evaluate the expression of PES1 in HNSCC cell lines and clinical tissues. PES1 was knocked down in TU177 and FaDu cells which have high PES1 expression. The effects of PES1 on cell proliferation and tumour growth in HNSCC were elevated by colony formation, CCK8 assays and tumorigenicity assay in nude mice. The effects on cisplatin (CDDP) sensitivity upon silencing of PES1 were assessed using a patient‐derived xenograft (PDX) model.

**Results:**

PES1 expression was an independent prognostic factor for HNSCC and negatively associated with the overall survival rate. Silencing of PES1 reduces HNSCC cell proliferation and tumour growth. Moreover, PES1 inhibition significantly sensitises HNSCC cells to cisplatin. Furthermore, we found a PES1 has a high correlation with c‐Myc and plays an essential role in the tumour immune microenvironment.

**Conclusion:**

Our findings suggest that PES1 is associated with tumour growth and drug resistance and served as a potential cancer marker for diagnosis and a putative therapeutic target for HNSCC.

## Graphical abstract

PES1 is associated with tumor growth and drug resistance, and served as a potential cancer marker for diagnosis and a putative therapeutic target for HNSCC.

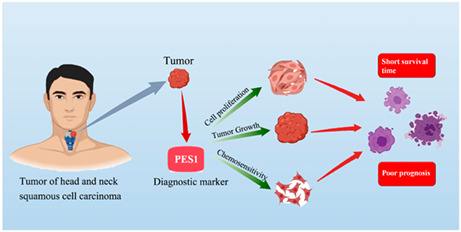

## 1 INTRODUCTION

As a common type of human cancer, head and neck squamous cell carcinoma (HNSCC) originates from the nasopharynx, lip, oral cavity, larynx, oropharynx and hypopharynx.^1^ Globally, approximately HNSCC accounts for an estimated 870,000 new cases and 444,000 deaths in 2020.^2^ Despite improved understanding and advances in chemotherapy, radiotherapy, surgery, targeted therapy and immunotherapy, HNSCC remains one of the most devastating cancers worldwide, with short survival times and poor prognosis.^3^, ^4^ Approximately, half of the patients with locally advanced HNSCC experience cancer recurrence and drug resistance after initial treatment, resulting in an incredibly poor prognosis.^5^, ^6^, ^7^ Currently, the pathogenesis and etiopathogenesis of HNSCC remain unclear. This prompted us to identify early diagnosis and novel therapeutic targets for HNSCC.

The pescadillo ribosomal biogenesis factor 1 (PES1, known as Pescadillo) gene is located on human chromosome 22q12.2 and includes 19 exons.^8^ As a nuclear protein encoder, PES1 is involved in tumour progression, cell senescence, cell cycle regulation, DNA replication and ribosome biogenesis.^8^, ^9^ Previous studies have reported that PES1 plays a critical role in the progression of various cancers, including gastric, breast, pancreatic, colorectal and hepatocellular carcinoma.^9^, ^10^, ^11^, ^12^, ^13^ In addition, the underlying molecular mechanism was preliminarily explored, and these studies mentioned potential relationships between PES1 and the PI3K/AKT pathway, HIF‐1α, JNK and c‐Myc.^9^, ^10^, ^12^, ^14^ However, the role of PES1 in HNSCC is unknown, and there is an urgent need to elucidate the function and underlying mechanisms of PES1 in HNSCC.

The chemotherapy drug, cisplatin, induced the apoptosis of cancer cells in solid tumours when it was first introduced in the 1960s.^15^ Cisplatin‐based chemotherapy for patients with locally advancing HNSCC typically provides an initial response rate of 50%.^16^ Most patients who receive cisplatin develop acquired resistance, which leads to cancer recurrence in half of the population. Among HNSCC patients, cisplatin resistance and cancer recurrence are the main causes of therapeutic failure in cisplatin therapy.^17^ Therefore, the search for combination regimens that increase cisplatin sensitivity is important for improving the clinical outcome of patients with HNSCC.

In the current study, using a combination of experimental and bioinformatics data, we found that PES1 is a novel diagnostic and prognostic biomarker for HNSCC. Silencing of *PES1* led to a reduction in HNSCC cell proliferation and tumour growth and increased HNSCC cell sensitivity to cisplatin. We further analysed the possible mechanisms and immune infiltration underlying PES1 actions in HNSCC.

## 2 MATERIALS AND METHODS

### 2.1 Data and HNSCC specimen acquisition

Data on HNSCC gene expression and associated clinical information were obtained from the TCGA database (http://www.cbioportal.org/). This data set is in transcripts per million (TPM) and normalised by log_2_(TPM + 1). The median expression value of *PES1* was used to classify patients with HNSCC into low and high expression groups. To independently validate the results from the Gene Expression Omnibus, two more microarray data sets were downloaded (GSE59102 and GSE127165). HNSCC tumour and adjacent normal mucosal (ANM) tissues were obtained from 12 patients who received surgery at the First Affiliated Hospital of Anhui Medical University (Anhui, China) between 2019 and 2020. Written informed consent was obtained from all the participants before participation. The study was approved by the Research Ethics Committee of the First Affiliated Hospital of Anhui Medical University.

### 2.2 Cell culture

HEK293T, Cal27 and FaDu cells were provided by the American Type Culture Collection. Tu177, HSC‐3, normal oral keratinocytes (NOK) and Tu212 cells were purchased from Otwo Biotech, Inc. Cells were incubated at 37°C with 5% CO_2_ and grown in DMEM or RPMI‐1640 containing 1% penicillin–streptomycin and 10% foetal bovine serum.

### 2.3 Western blot

RIPA lysis buffer was used to extract total cellular protein (Beyotime Biotechnology). Protein expression was determined by western blot as previously described.^18^ Briefly, lysates were separated from cells or tissues using NuPAGE 4%–12% Bis‐Tris and transferred to PVDF membranes (Millipore). Non‐fat milk (5%) was used to block the bands before the primary antibodies were applied. After incubation overnight at 4°C with primary antibodies and the secondary antibody for 1 h, the results were obtained using chemiluminescence. The following commercial antibodies were used: anti‐PES1 (ab252849, Abcam), anti‐beta actin (ab8226, Abcam) and anti‐GAPDH (ab8245, Abcam).

### 2.4 Lentiviral infection

HNSCC cells (Tu177 and FaDu) were transfected with short hairpin RNAs (shRNAs) targeting PES1 using lentivirus vector GV248 (GenePharma). The target sequences used were shown as followed: shPES1#1: 5′‐GAGGCCTTGAGAAGAAGAAGT‐3′, shPES1#2: 5′‐TAGAGCGTTTAAAGGACAATA‐3′ and shSc, 5′‐TTCTCCGAACGTGTCACGT‐3′. HEK293T cells were co‐transfected with these recombinant plasmids and packing vectors (pVSVG and psPAX2). After transfection of 48–72 h, the viral supernatants were collected and filtered for HNSCC cell infection. We then infected the cells with lentivirus for 24 h in the presence of polybrene (8 mg/ml) and selected them with puromycin (10 mg/ml).

### 2.5 Histology and immunohistochemistry (IHC)

In all specimens, 4% formalin was used for 24 h for fixation, paraffin was applied and 4 mm sections were cut. Immunohistochemical staining was performed using the following primary antibodies after deparaffinisation: PES1 (NB110‐40548, NOVUS, diluted 1:50) and Ki‐67 (Cat# 9027S, Cell Signalling Technology, diluted 1:100).

### 2.6 EdU incorporation assay

Tu177 and Fadu cells (5 × 10^4^ cells/well) were seeded in a 24‐well plate. A Cell‐Light EdU DNA Cell Proliferation Kit (RiboBio) was used to perform the EdU assay. After that, each well was incubated with 50 μM EdU for another 2 h. Then, the cells were fixed, stained and photographed.

### 2.7 Immunofluorescence (IF) staining

TU177 and FaDu cells with or without PES1 knockdown were grown on 24 coverslips. A 15‐min fixation with 4% paraformaldehyde, permeabilization with 0.5% Triton X‐100 and blocking with 3% bovine serum albumin were performed. A PES1‐specific antibody was incubated at 4°C overnight with cells. The coverslips were stained with DAPI after incubation with Alexa Fluor 488 goat anti‐rabbit IgG (A‐11006; Thermo Fisher Scientific). Fluorescent images were captured by using an LSM880 + Airyscan confocal laser scanning microscope (Carl Zeiss).

### 2.8 Quantitative real‐time PCR (qRT‐PCR)

According to the manufacturer's instructions, TRIzol (Life Technologies) was used to extract RNA from the cells. To reverse‐transcribe RNA into cDNA, we used the TaKaRa PrimeScript™ RT Reagent Kit. Five‐fold dilutions of cDNA were used as templates for quantitative real‐time PCR. Life Technologies Power SYBR® Master Mix was used to perform qRT‐PCR, according to the manufacturer's protocol. To detect PES1, oligonucleotide primers were synthesised using β‐actin as the internal control. The following primer sequences were used: human PES1 forward, 5′‐CTTTGTCAACTTCCGCCTTTAC‐3′, and reverse, 5′‐AGTTTCTCCATACAACTCTCGG‐3′; β‐actin forward, 5′‐CTGGCACCACACCTTCTACAATG‐3′ and reverse 5′‐GGCGTACAG GGATAGCACAGC‐3′.

### 2.9 Cell proliferation assay

According to the manufacturer's instructions, the CCK8 assay was used to monitor cell proliferation and viability (Beyotime Shanghai). Each experiment was independently performed in triplicate. Briefly, cells were seeded at a density of 1000 cells/well into 96‐well plates and cultured in normal growth conditions for 24 h. Ten microlitres of CCK8 solution were added to each well at a specified time. The 450‐nm absorbance was measured using a multi‐scan spectrophotometer 3 h later.

### 2.10 Colony formation assay

We plated cells in 6 cm cell culture plates at 1000 cells/well and cultured them for 2 weeks at 37°C in a tissue fixative solution before staining them with 0.1% crystal violet. We numbered each colony of cells and repeated the experiment thrice.

### 2.11 Animal experiments and patient‐derived xenograft (PDX) models

Mice used in the experiments (4‐week‐old male NOD/SCID mice and BALB/c nude mice) were purchased from GemPharmatech Co., Ltd. HNSCC cells (5 × 10^6^) were subcutaneously inoculated into each mouse (five per group) for tumorigenicity assays. Using the formula *V* = 0.5 * *W* ^2^ × *L* (where *V* represents volume, *L* represents length and W represents width), the tumour volume was calculated.

Fresh tumour tissues for PDX models were obtained from patients with laryngeal squamous cell carcinoma (LSCC). Tissues were processed, and 2 × 2 × 3‐mm^3^ tumour pieces were grafted into the dorsal flanks of NOD/SCID mice as P0. Tumours from PDX mice were harvested when they reached 1000 mm^3^ and passaged into another batch of mice, named P1. Subsequently, PDX tumours were transplanted into next‐generation mice (P2). They were then randomly divided into four groups when the tumour could detectable (five per group) and treated with the combination treatment of normal saline (NS), cisplatin (3 mg/kg, twice per week), non‐targeting control siRNAs (100 μg, twice per week) or PES1 siRNAs (100 μg, twice per week). Two or more injections of siRNA were administered to the tumour bodies at a time. Concurrently, the mice were challenged intraperitoneally with saline or cisplatin. This treatment continued for 3 weeks. Chemically modified siRNAs from GenePharma were used for intratumoural injections. The target sequences were shown as follows: siNC:5′‐UUCUCCGAACGUGUCACGUTT‐3′, siPES1‐1:5′‐GGCCUUGAGAAGAAGAAGUTT‐3′ and siPES1‐2:5′‐GAGCGUUUAAAGGACAAUATT‐3′. Transfection reagents for in vivo were purchased from Engreen Biosystem Co., Ltd. (Beijing, China).

### 2.12 Bioinformatics and statistical analysis

Kaplan–Meier (K–M) survival curves were generated using the survminer and compared using a log‐rank test between subgroups. We evaluated the diagnostic accuracy of PES1 using the receiver operating characteristic (ROC) curve and the area under the ROC curve (AUC). PES1‐associated genes (Pearson correlation analysis) were collected from the TCGA data sets. An enrichment analysis using Metascape online software was performed on functional hallmark pathways with a correlation greater than or equal to 0.3 with PES1. Gene set enrichment analysis (GSEA) was used to determine the degree of Hallmark MYC targets V1 pathway enrichment between the two groups with high and low *PES1* expression in nine cancers. To explore the correlation between PES1 and infiltrating immune cells, CIBERSORT was used to determine the proportion of 22 different infiltrating immune cells in the tumour immune microenvironment. Additionally, we examined the specific differences between PES1 and key immune checkpoints. The R package estimate was used to analyse the tumour ESTIMATE score, immune score, stromal score and tumour purity.

Statistical analyses were performed using R software and GraphPad Prism 6.0. Student's *t* tests were used to examine the differences between the two experimental groups. Pearson's correlation coefficient (*r*) was used to analyse the correlation between the variables and to present the results. Statistical significance was set at *p* < 0.05 (*p < 0.05, **p < 0.01 and ***p < 0.001).

## 3 RESULTS

### 3.1 PES1 is a novel diagnostic and prognostic biomarker of HNSCC

**FIGURE 1:**
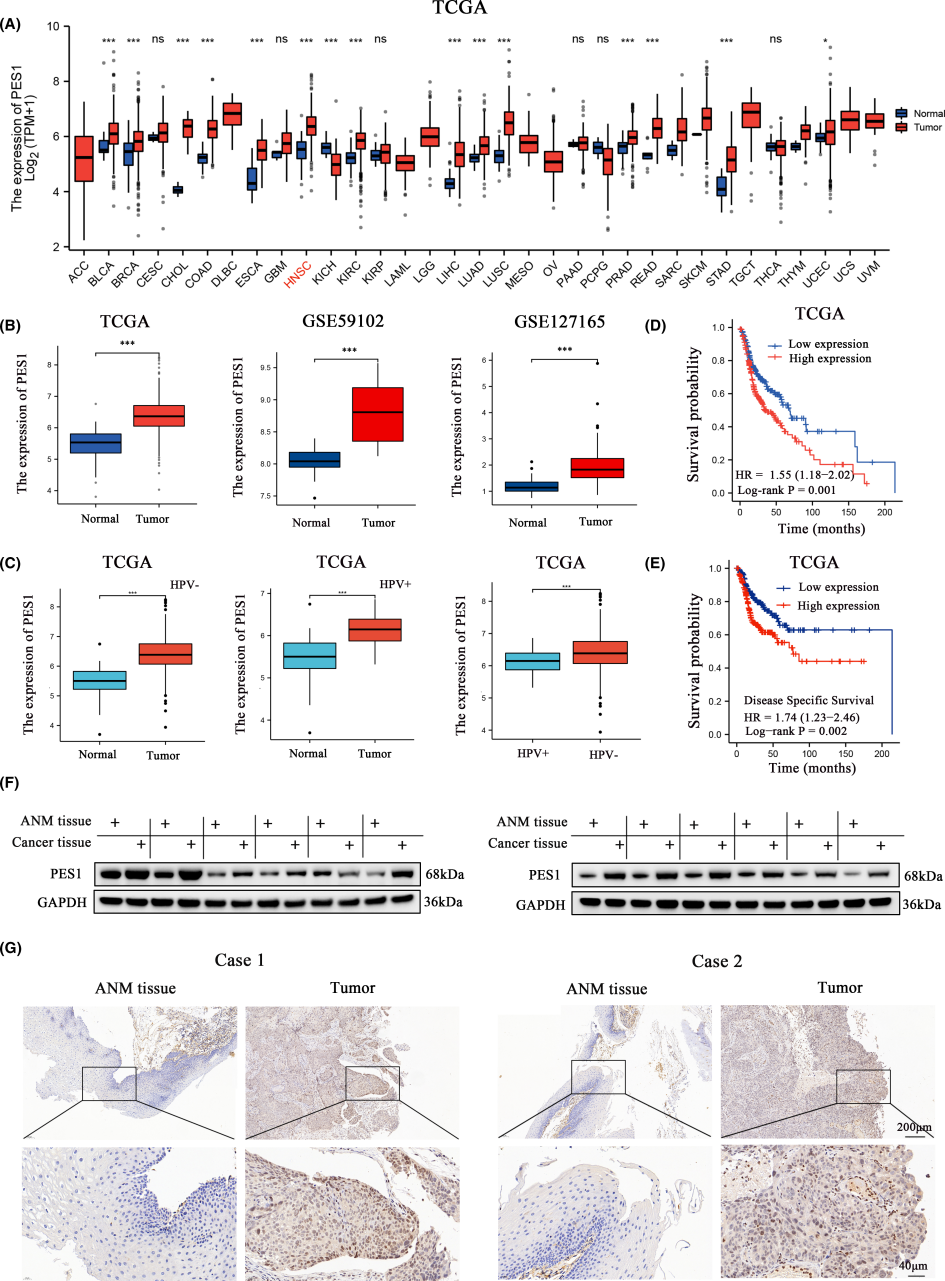
As a new diagnostic biomarker, PES1 is highly expressed in head and neck squamous cell carcinoma (HNSCC) and correlates with poorer prognosis. (A) Pan‐cancer expression of PES1 between tumour tissues and normal tissues from TCGA database. (B) The expression of PES1 in normal tissues and tumour tissues was obtained from the TCGA GSE59102 and the GSE127165 data sets. (C) The relationship between PES1 expression levels and HPV status. (D, E) Kaplan–Meier plot for overall survival (D) and disease‐specific survival (E) of HNSCC patients in TCGA data set based on PES1 expression. (F, G) PES1 protein expression levels in the paired HNSCC tissues and ANM tissues using western blot (F) and immunohistochemistry (G) analyses. Scale bar, low magnification: 200 μm, high magnification: 40 μm

A pan‐cancer analysis of the TCGA database showed that PES1 is highly expressed in various types of cancer, such as bladder urothelial carcinoma (BLCA), HNSCC, cholangiocarcinoma (CHOL), lung adenocarcinoma (LUAD), oesophageal carcinoma (ESCA), breast invasive carcinoma (BRCA), kidney renal clear cell carcinoma (KIRC) and colon cancer (COAD), etc. (Figure 1A). These results are partly in accord with the findings of previous studies.^12^ However, few studies have explored the relationship between PES1 and HNSCC. TCGA, GSE59102 and GSE127165 databases revealed that PES1 was highly expressed in HNSCC tissues (Figure 1B). In addition, we compared the differences in PES1 expression levels in HPV‐negative cancer and normal tissues, and the results showed that PES1 was highly expressed in tumour tissues (Figure 1C). Similar to the results in the HPV‐negative group, PES1 was highly expressed in HPV‐positive tumour tissues (Figure 1C). We then compared the results by grouping according to HPV, and PES1 was expressed at higher levels in the HPV‐negative group, and the differences were statistically significant (Figure 1C). Finally, as previously reported, the survival time of patients with HPV‐negative HNSCC was shorter than that of patients in the HPV‐positive group (Figure S1B). We compared the survival difference between patients with high and low expression of PES1 in HPV‐positive subgroups, the results showed that patients with high expression of PES1 had a poor prognosis (Figure S1C). In the HPV‐negative group, compared with the low expression group, the prognosis of patients with high expression of PES1 was worse, but the difference was not statistically significant (Figure S1D). ROC curve analysis was performed using TCGA (AUC:0.908), GSE59102 (AUC:0.947) and GSE127165 (AUC:0.887) databases, and results suggest that PES1 may be a good diagnostic marker for HNSCC (Figure S1A). Additionally, PES1 expression was an independent prognostic factor for overall survival (OS) and disease‐specific survival (DSS) of HNSCC patients (Tables 1 and 2) and was negatively correlated with the OS and DSS (Figure 1D,E).

**TABLE 1:** Univariate and multivariate Cox regression analyses of the correlation between clinical characteristics with overall survival

Characteristics	Total (*N*)	Univariate analysis		Multivariate analysis	
		Hazard ratio (95% CI)	*p* value	Hazard ratio (95% CI)	*p* value
Age	499				
<=60	244	Reference			
>60	255	1.238 (0.945–1.621)	0.122	1.208 (0.909–1.604)	0.192
Gender	499				
Female	133	Reference			
Male	366	0.754 (0.566–1.004)	0.054	0.740 (0.547–1.003)	0.052
Histologic grade	480				
G1&G2	359	Reference			
G3&G4	121	0.942 (0.690–1.287)	0.709	0.950 (0.694–1.301)	0.751
Clinical stage	485				
I‐II	113	Reference			
III‐IV	372	1.214 (0.875–1.683)	0.245	1.220 (0.870–1.710)	0.249
PES1	499				
Low	250	Reference			
High	249	1.551 (1.183–2.033)	**0.002**	1.524 (1.153–2.016)	**0.003**

The bold values are defined as significant (*p* < 0.05).

**TABLE 2:** Univariate and multivariate Cox regression analyses of the correlation among the clinical characteristics with disease‐specific survival

Characteristics	Total (*N*)	Univariate analysis		Multivariate analysis	
		Hazard ratio (95% CI)	*p* value	Hazard ratio (95% CI)	*p* value
Age	474				
<=60	234	Reference			
>60	240	1.058 (0.748–1.497)	0.750	0.998 (0.698–1.426)	0.991
Gender	474				
Female	122	Reference			
Male	352	0.956 (0.643–1.421)	0.825	0.895 (0.594–1.347)	0.594
Histologic grade	460				
G1&G2	344	Reference			
G3&G4	116	1.059 (0.717–1.564)	0.772	1.068 (0.721–1.582)	0.743
Clinical stage	460				
Stage I&Stage II	106	Reference			
Stage III&Stage IV	354	1.143 (0.747–1.749)	0.537	1.123 (0.726–1.736)	0.602
PES1	474				
Low	240	Reference			
High	234	1.752 (1.228–2.500)	**0.002**	1.678 (1.171–2.406)	**0.005**

The bold values are defined as significant (*p* < 0.05).

Furthermore, western blot (Figure 1F) and IHC (Figure 1G) analyses revealed that PES1 was substantially overexpressed in HNSCC tissues compared to the corresponding ANM tissues. Taken together, these data suggest that PES1 is a novel diagnostic and prognostic biomarker for HNSCC.

### 3.2 Construction of the PES1‐manipulated HNSCC cell lines

**FIGURE 2:**
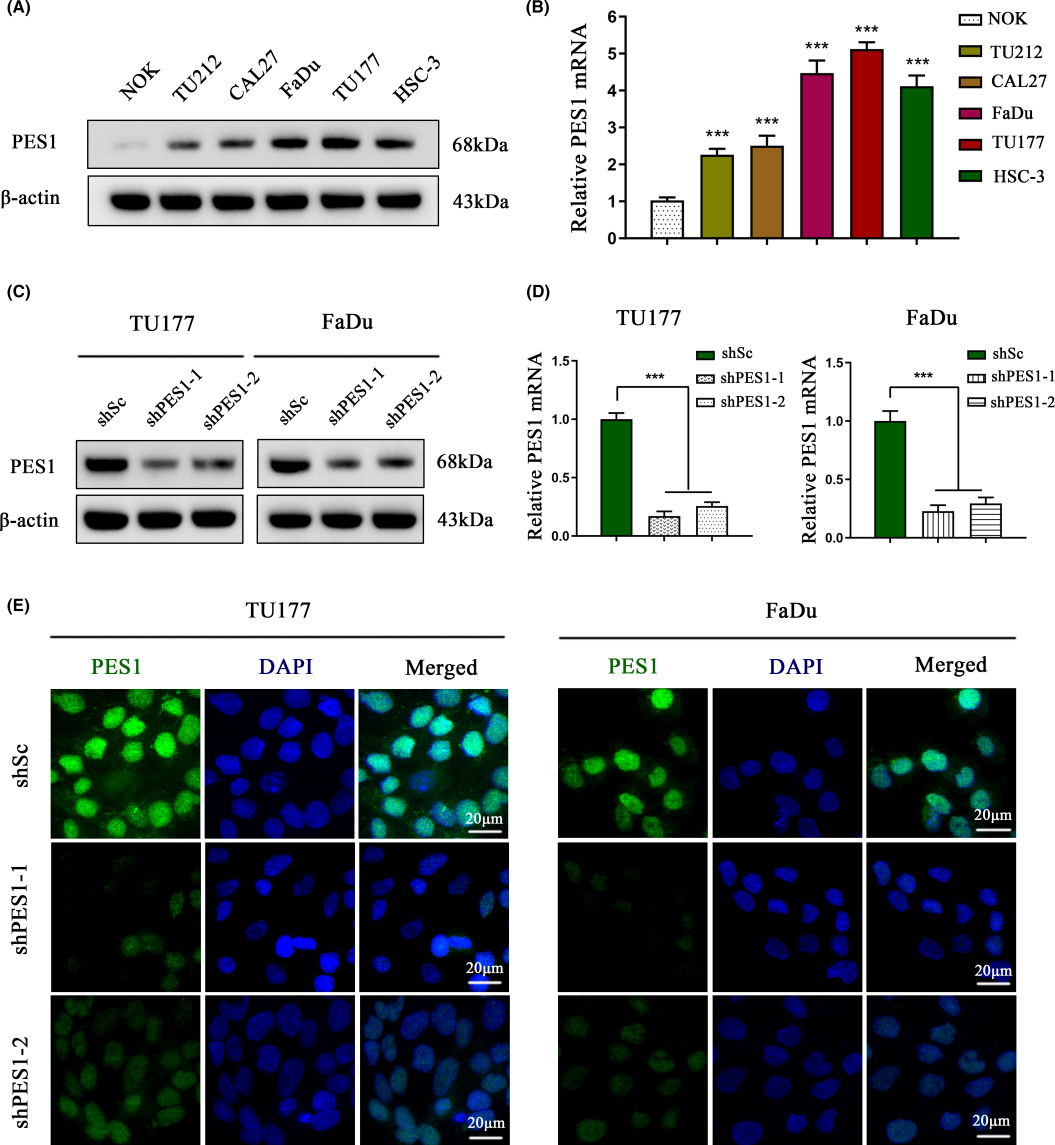
Construction of the PES1‐manipulated head and neck squamous cell carcinoma (HNSCC) cell lines. (A, B) The protein level (A) and mRNA expression (B) of PES1 in normal oral keratinocytes and five HNSCC cell lines. (C–E) Examination of stable knockdown of PES1 in TU177 and FaDu cells. Western blot (C) analysis, qRT‐PCR (D) analysis, immunofluorescence staining (E). ***p < 0.001.

To further elucidate the function of PES1, we examined PES1‐regulated HNSCC cells. First, PES1 expression was assessed in NOK and HNSCC cell lines (TU212, CAL27, FaDu, TU177 and HSC‐3). Owing to the relatively high expression of PES1 in TU177 and FaDu cells (Figure 2A,B), we established stable PES1‐knockdown cell lines to determine the biological functions of PES1 in HNSCC. The efficacy of PES1 knockdown in these cell lines was verified by western blot and qPCR (Figure 2C,D). The nuclear localisation and decreased expression of PES1 in response to shRNA‐expressing lentivirus were corroborated by IF (Figure 2E).

### 3.3 Silencing of PES1 reduces HNSCC cell proliferation and tumour growth

**FIGURE 3:**
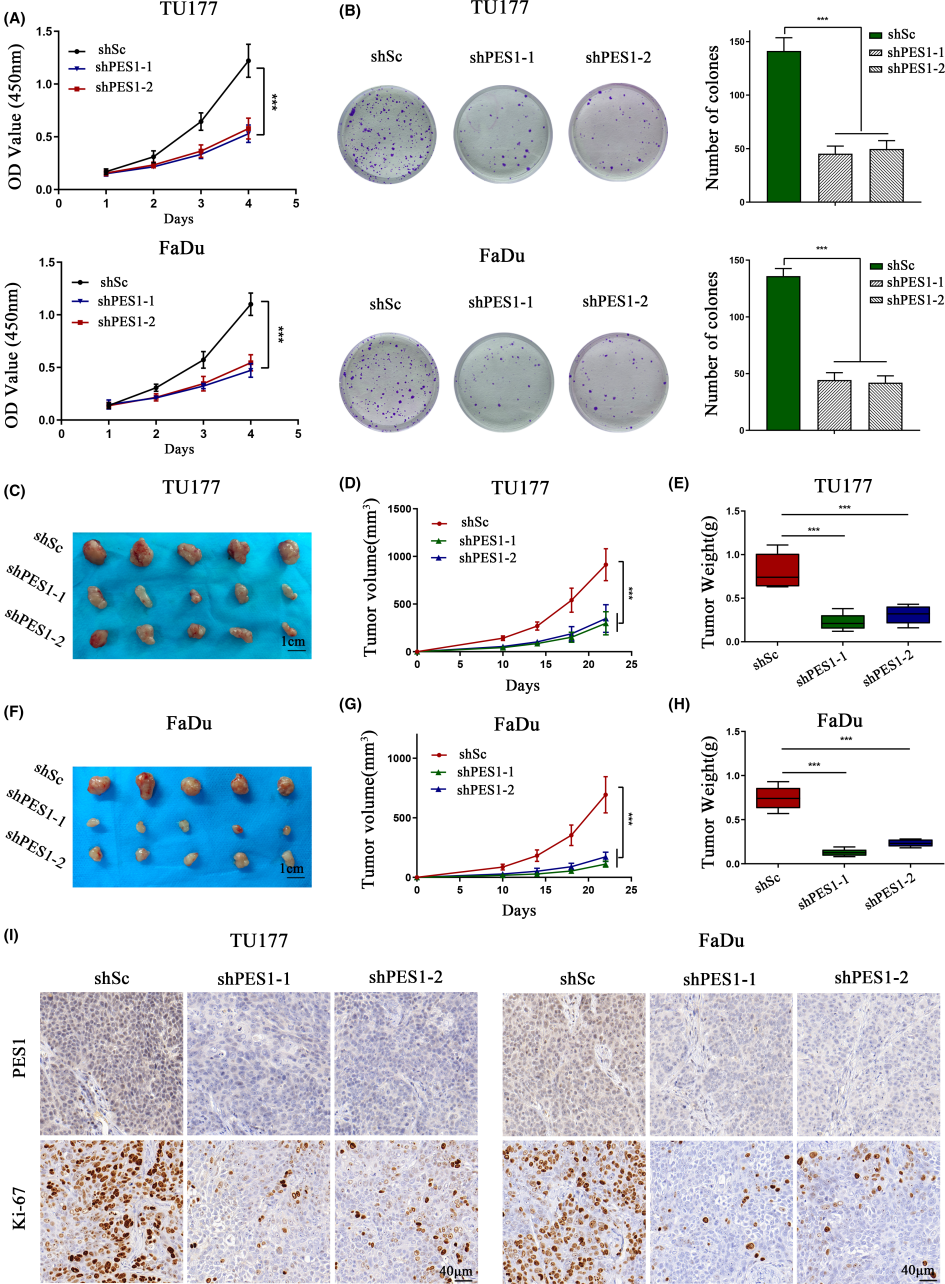
PES1 promotes head and neck squamous cell carcinoma cell proliferation and tumour growth. (A–I) TU177 and FaDu cells were transduced with PES1 shRNAs‐expressing (shPES1‐1 or shPES1‐2) lentiviruses or scrambled control (shSc) lentiviruses. (A, B) CCK8 (A) and colony formation (B) were used to evaluate cellular proliferation in vitro. Error bars represent mean ± SD for triplicate experiments. ***p < 0.001. (C–I) The indicated UT177 and FaDu cells were inoculated subcutaneously into the right flank of nude mice and the tumour growth was measured after the inoculation. Tumour images (C, F), tumour growth curves (D, G), tumour weight (E, H) and representative IHC images of PES1 and Ki‐67 (I). Scale bars, 1 cm (C, F), 40 μm (I).

CCK8 and colony formation assays indicated that the knockdown of PES1 significantly reduced HNSCC cell proliferation and colony formation (Figure 3A,B). We further validated the tumour‐promoting effects of PES1 in HNSCC cells. TU177 and FaDu cells with or without PES1 knockdown were subcutaneously injected into nude mice. The results show that silencing of ITGA5 significantly decreases the tumorigenic capacity of TU177 and FaDu cells compared to the corresponding control cells (Figure 3C–H). In addition, the EdU incorporation test showed that the number of EdU‐positive cells in PES1 knockdown cells was significantly reduced (Figure S2A,B). Moreover, the expression levels of Ki‐67 (a key indicator of cell growth) were clearly reduced in tumour tissues with the deletion of PES1 (Figure 3I). In summary, PES1 promotes HNSCC cell proliferation and tumour growth in vivo and in vitro.

### 3.4 Depletion of PES1 increases HNSCC cell sensitivity to cisplatin in PDX models

**FIGURE 4:**
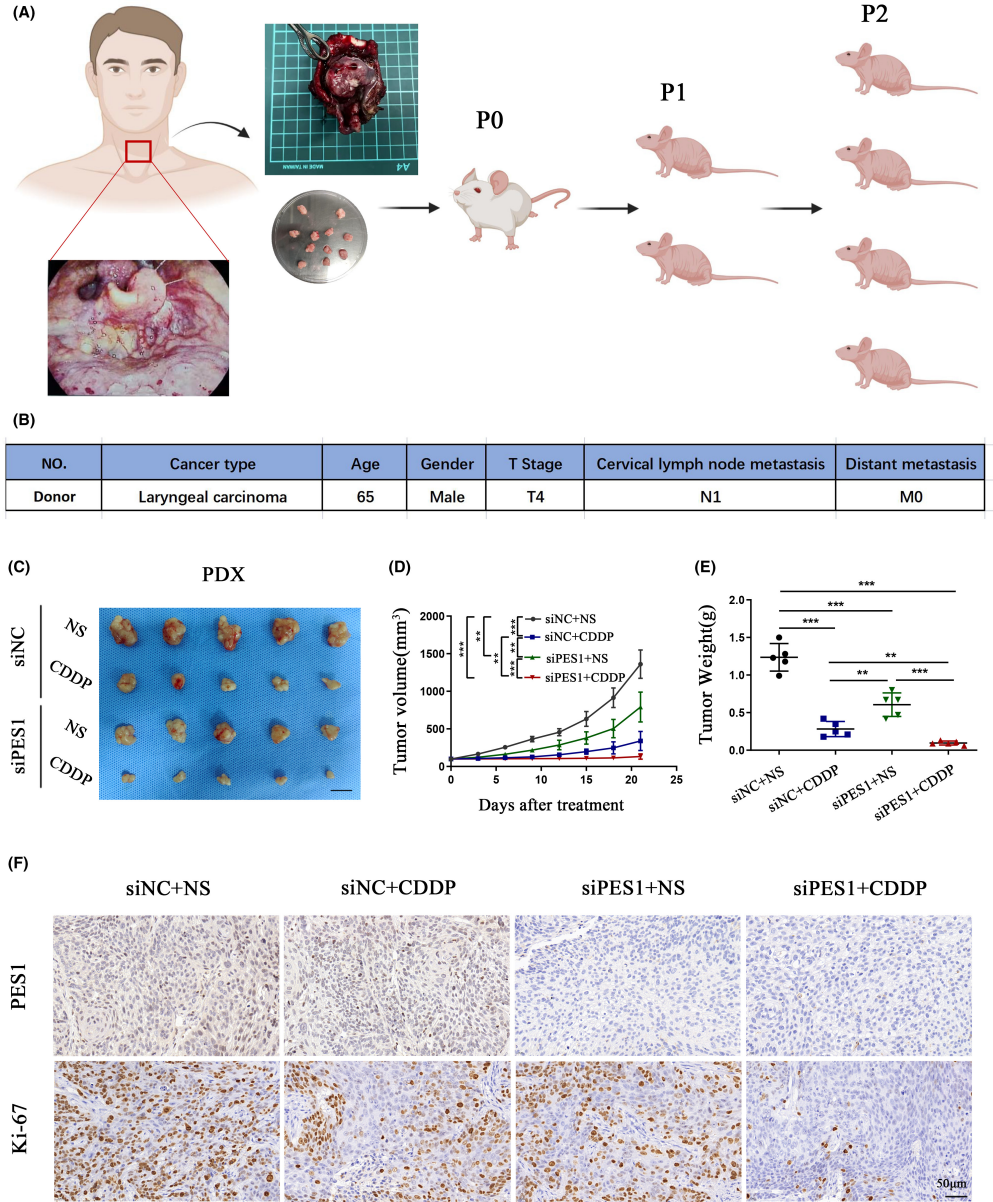
Depletion of PES1 increases head and neck squamous cell carcinoma cell sensitivity to CISPLATIN in PDX models. (A) Schematic flow chart of HNSCC PDX model (image partly retrieved from https://app.biorender.com/biorender‐templates). (B) Clinical information of the donor. (C–F) Twenty PDX models of P2 were randomly divided into four groups and treated with different combinations as indicated. Images of the tumour (C), tumour growth curves (D), tumour weight (E) and representative immunohistochemistry images staining for PES1 and Ki‐67 (F).

We further investigated the clinical role of PES1 in HNSCC by determining whether PES1 alters HNSCC sensitivity to cisplatin, the most common chemotherapeutic drug for HNSCC.^19^ First, a PDX HNSCC model was established. As shown in Figure 4A,B, fresh tumour tissues were isolated from a 65‐year‐old patient with laryngeal squamous cell carcinoma. The tissues were grafted subcutaneously into the flank of immunodeficient mice to establish PDX models. Results show that administration of PES1 siRNA or intraperitoneal injection of cisplatin inhibited tumour progression in HNSCC. However, the combination treatment group exhibited better therapeutic effects (Figure 4C–E). IHC staining showed that the expression level of Ki‐67 was decreased in the treatment groups compared to the control subjects and markedly reduced in response to the combination treatment (Figure 4F). Collectively, the silencing of PES1 increased chemosensitivity to cisplatin in HNSCC cells.

### 3.5 Identifying PES1‐impacted signalling pathways

**FIGURE 5:**
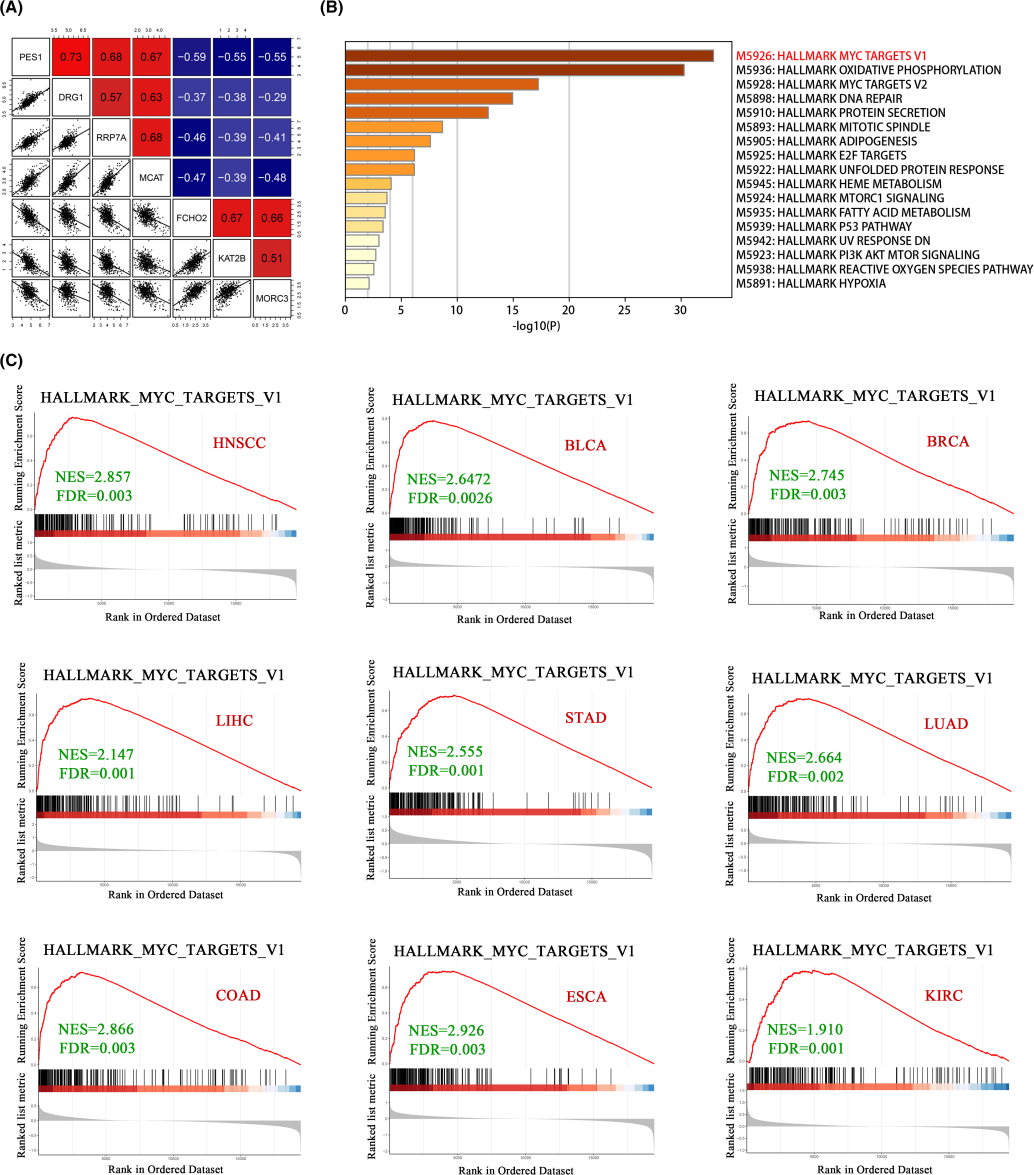
PES1 may play a role in promoting the proliferation of head and neck squamous cell carcinoma (HNSCC) through the MYC targets V1 signalling pathway. (A) The correlation between PES1, DRG1, RRP7A, MCAT, FCHO2, KAT2B and MORC3 expression of HNSCC in the TCGA cohort. (B) The hallmark analysis of PES1‐related genes using the Metascape database. (C) Gene set enrichment analysis (GSEA) plot of MYC targets the V1 signal pathway in multiple cancers.

To specify the signalling pathways involved in the role of PES1 in promoting HNSCC proliferation, we first screened the TCGA data set for genes with a correlation of 0.3 and above and identified a total of 2356 genes (Table S1). As shown in Figure 5A, the top three genes had the highest positive and negative correlations with PES1 expression. We then performed enrichment analysis on all the above genes using Metascape. The results showed that the enriched pathways were mainly MYC targets V1, oxidative phosphorylation, MYC targets V2, DNA repair and protein secretion (Figure 5B). Finally, we used GSEA to analyse the association of PES1 with the MYC targets V1 pathway in nine cancer types, including HNSCC. As expected, MYC targets V1 were enriched in patients with high PES1 expression (Figure 5C).

### 3.6 The relationship between PES1 expression level to immune microenvironment and immune checkpoints in TCGA data set

**FIGURE 6:**
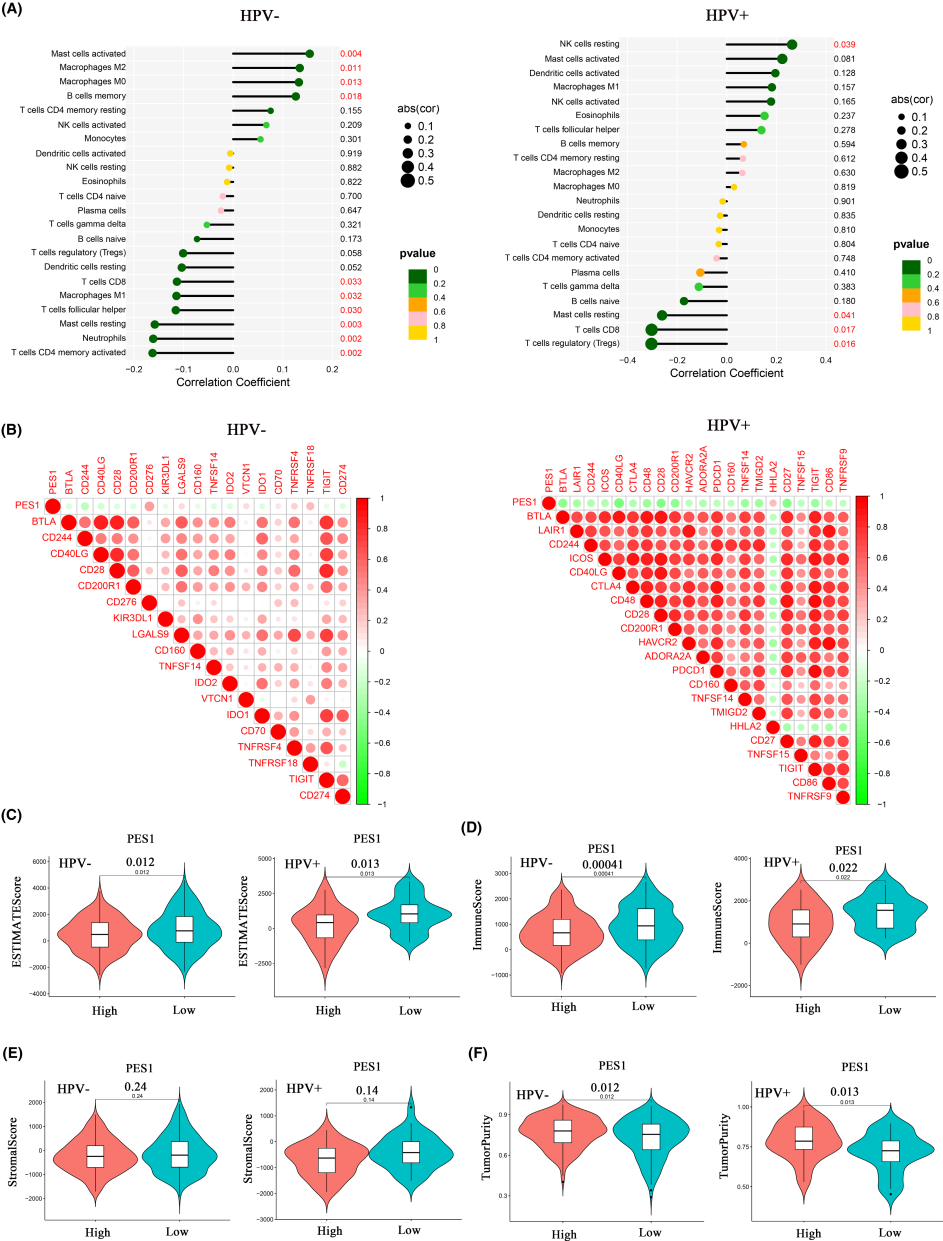
Relationship between PES1 expression and the abundance of tumour immune infiltrating cells, immune checkpoint genes and tumour microenvironment under different HPV statuses. (A) Correlation between PES1 expression and tumour immune infiltrating cells in HPV‐negative (left panel) and HPV‐positive groups (right panel). (B) There was an association between PES1 expression and immune checkpoint genes in HPV‐negative (left panel) and HPV‐positive groups (right panel). (C–F) Differences in ESTIMATE score (C), immune score (D), stromal score (E) and tumour purity (F) between PES1 high and low expression groups.

Given the important role of the microenvironment in the malignant biological behaviour of tumours, we analysed the correlation between PES1 expression levels and the abundance of immune infiltrating cells in HPV‐negative and HPV‐positive groups, respectively. As shown in Figure 6A, in the HPV‐negative subgroup, the expression level of PES1 was positively correlated with the abundance of Mast cells activated, Macrophages M2, macrophage M0 and B cells memory and negatively correlated with the abundance of T cells CD4 memory activated, Neutrophils, Mast cells resting and T cells follicular helper, etc. In the HPV‐positive subgroup, PES1 expression levels were positively correlated with the abundance of NK cells resting and negatively correlated with the abundance of T cells regulatory (Tregs), T cells CD8 and Mast cells resting (Figure 6A). Immunotherapy has become a systematic treatment method to improve the prognosis of advanced cancer. Therefore, we analysed the correlation between PES1 and multiple common immune checkpoint proteins as a guide for therapy. PES1 was negatively associated with CD244, CD40LG and IDO2 immune checkpoint genes but positively associated with TNFRSF18 and CD276 in the HPV‐negative group (Figure 6B). PES1 was negatively associated with most immune checkpoint genes in the HPV‐positive group, which was different from the HPV‐negative group (Figure 6B). In addition, we analysed the relationship between PES1 and ESTIMATE score, immune score, stromal score and tumour purity in different HPV subgroups to elucidate the potential association between the expression level of this gene and the tumour microenvironment. The results showed that patients in the PES1 low expression group had higher ESTIMATE scores and immune ratings and lower tumour purity, regardless of HPV status (Figure 6C,D,F). However, there were no significant differences in PES1 expression levels and stromal scores between HPV‐negative and ‐positive patients (Figure 6E).

## 4 DISCUSSION

As an important tumour‐promoting factor, PES1 plays an important role in tumour development and progression. For instance, PES1 has been shown to promote cell proliferation and is upregulated in neuroblastoma and ovarian cancer.^20^, ^21^ Qiu et al. found that PES1 promotes papillary thyroid cancer cell migration and invasion.^22^ In this study, using the TCGA database, pan‐cancer analysis was done. It was found that *PES1* expression is elevated in many cancers, including BLCA, BRCA, CHOL, COAD, ESCA, HNSCC, KIRC and LUAD. Comprehensive bioinformatics analysis identified *PES1* expression as an independent prognostic factor for HNSCC and negatively correlated with the overall survival rate. In follow‐up experiments, we knocked down *PES1* in HNSCC cells with high PES1 expression and found that silencing of *PES1* led to a reduction in HNSCC cell proliferation and tumour growth. Thus, as a novel diagnostic and prognostic biomarker, PES1 promotes tumour progression in HNSCC.

Cisplatin is the first‐line treatment for HNSCC and is often used for chemotherapy administered concurrently with other chemotherapeutic agents.^23^, ^24^, ^25^ Drug resistance is a common drawback of cancer chemotherapy and cisplatin is no exception.^26^ Thus, further optimisation of the combined cisplatin chemotherapy regimen is required. Jin et al. found that increased PES1 expression can lead to chemotherapy drug resistance in pancreatic cancer.^12^ We speculate whether the combination of PES1 with cisplatin may achieve better therapeutic effects. To simulate in vivo conditions, we established a PDX model by subcutaneous engraftment of HNSCC organoids into immunodeficient mice. As shown in Figure 4, results show that the combination treatment group (administration of PES1 siRNA and intraperitoneal injection of cisplatin) have better therapeutic effects.

Previous studies have demonstrated that PES1 may serve as a potential cancer marker for diagnosis or as a putative therapeutic target in cancer treatment.^27^ Several studies have explored the molecular mechanisms underlying the abnormal expression of PES1 during tumour progression in human cancers. For instance, Wang et al. found that PES1 is involved in cell proliferation and tumorigenesis of hepatocellular carcinoma by regulating the PI3K/AKT pathway.^14^ Alternatively, it has been shown that PES1 promotes breast cancer by regulating the balance between ERα and ERβ.^11^ Furthermore, some studies have shown that PES1 affects tumour progression by regulating HIF‐1α or JNK.^9^, ^10^ In this study, we first analysed the correlation between *PES1* and other genes in HNSCC using TCGA data sets and identified 2356 genes (with correlation coefficients greater than 0.3). Further analysis revealed that the genes with high PES1 correlation were mainly enriched in MYC targets V1 pathways, which is consistent with a previous study.^12^ As summarised in Figure 5C, further GESA analyses of multiple cancers yielded consistent results. In addition, we found that PES1 plays an essential role in the tumour immune microenvironment. However, limitations arise as these preliminary findings have not been experimentally confirmed.

## 5 CONCLUSION

In conclusion, we found that *PES1* expression was an independent prognostic factor for HNSCC and was negatively correlated with the overall survival rate. *PES1* knockdown significantly reduced HNSCC cell proliferation and tumour growth. Subsequently, silencing of *PES1* increased the chemosensitivity of HNSCC cells to cisplatin. Furthermore, we found a high correlation between PES1 and c‐Myc. Moreover, our study reveals that PES1 plays an essential role in the tumour immune microenvironment. Last, the results of our study suggest that PES1 is a potential cancer marker for diagnosis and a putative therapeutic target in HNSCC.

## AUTHOR CONTRIBUTIONS

**Dapeng Li:** Data curation (lead); formal analysis (equal); investigation (supporting); methodology (equal); resources (equal); software (lead); validation (lead); writing – original draft (lead). **Changyu Yao:** Data curation (equal); formal analysis (lead); investigation (lead); resources (lead); software (equal); supervision (supporting); validation (equal); writing – original draft (equal); writing – review and editing (equal). **Zhao Ding:** Data curation (equal); formal analysis (lead); funding acquisition (supporting); investigation (equal); methodology (equal); project administration (supporting); software (equal); supervision (lead); writing – original draft (equal); writing – review and editing (equal). **Ping Liu:** Conceptualization (supporting); data curation (equal); formal analysis (supporting); investigation (equal); methodology (supporting); project administration (supporting); supervision (supporting); writing – original draft (supporting); writing – review and editing (equal). **Xue Chen:** Data curation (equal); formal analysis (supporting); investigation (supporting); methodology (equal); resources (supporting); visualization (equal). **Weiwei Liu:** Data curation (supporting); formal analysis (supporting); investigation (equal); methodology (equal); project administration (equal). **Fangzheng Yi:** Data curation (equal); formal analysis (supporting); methodology (supporting); resources (equal); supervision (equal). **Chuanya Jiang:** Data curation (supporting); formal analysis (equal); investigation (equal); software (supporting); writing – original draft (supporting); writing – review and editing (supporting). **Hongwu Li:** Funding acquisition (equal); supervision (equal); writing – review and editing (supporting). **Yehai Liu:** Conceptualization (equal); funding acquisition (equal); project administration (equal); supervision (equal); writing – review and editing (equal). **Jing Wu:** Conceptualization (lead); funding acquisition (lead); project administration (equal); supervision (lead); writing – review and editing (lead).

## FUNDING INFORMATION

This work was supported by the Natural Science Foundation of China (grant no. 82171127), the Key Program in the Youth Elite Support Plan in Universities of Anhui Province (gxyqZD2017022), the Natural Science Foundation of Universities of Anhui Province (KJ2019A0219), the Natural Science Research Project of Anhui Higher Education Institution (KJ2018ZD021) and discipline construction project of the First Affiliated Hospital of Anhui Medical University (no. 4245).

## CONFLICT OF INTEREST

The authors declare that they have no conflict of interest.

## Supporting information


Figure S1

Figure S2

Table S1

## Data Availability

The data sets generated and/or analysed during the current study are available from the corresponding author upon reasonable request.
