# Supplementary figures and images for: Pescadillo ribosomal biogenesis factor 1 reduction suppresses tumour growth and renders chemosensitivity of head and neck squamous cell carcinoma
Source: Cancer Med. 2022 Oct 11;12(5):5703–17. doi: 10.1002/cam4.5315 (PMC10028059; doi:10.1002/cam4.5315)

**A****TCGA**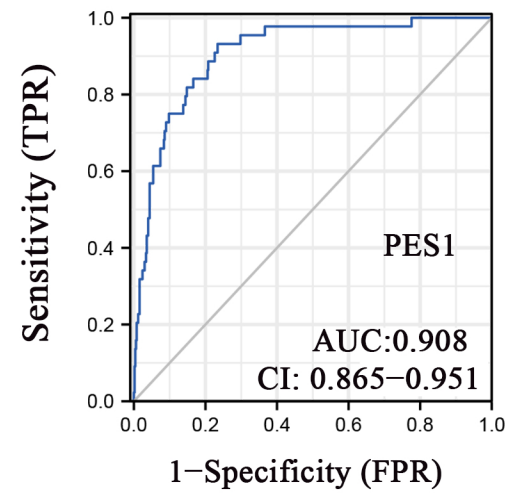**GSE59102**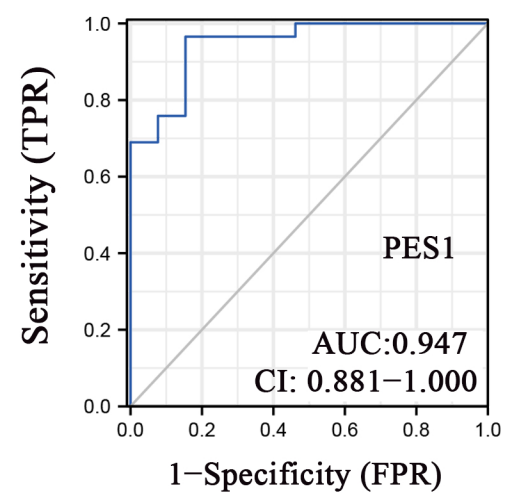**GSE127165**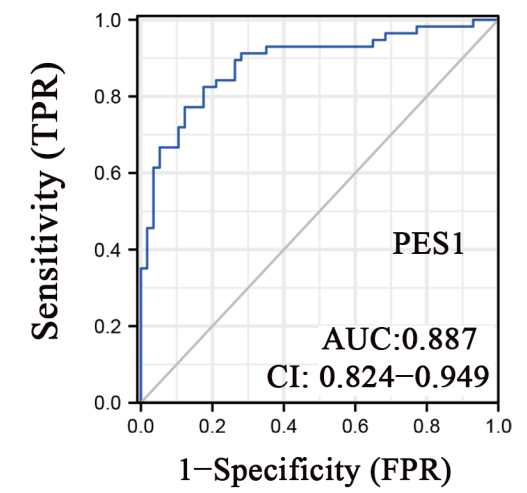**B****TCGA**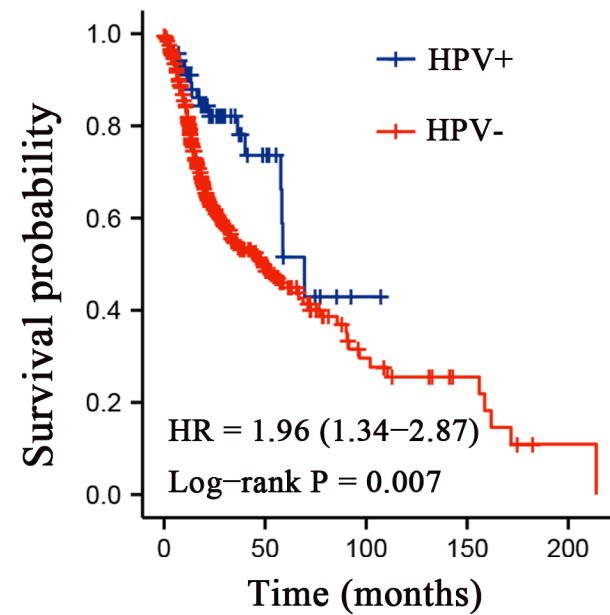**C****TCGA**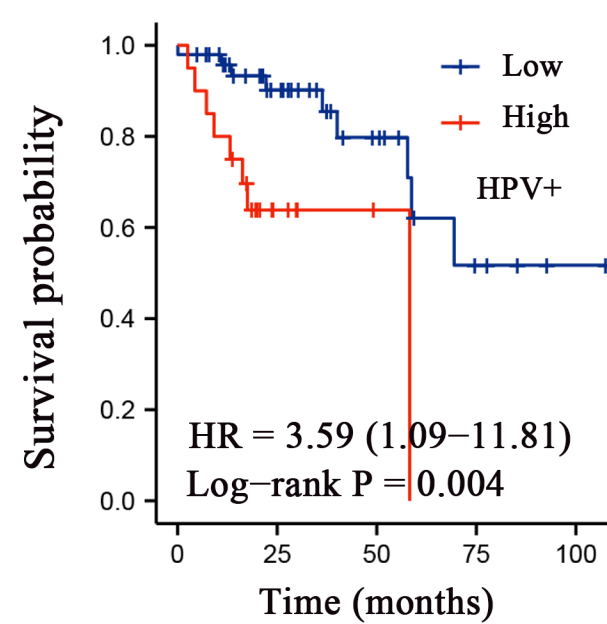**D****TCGA**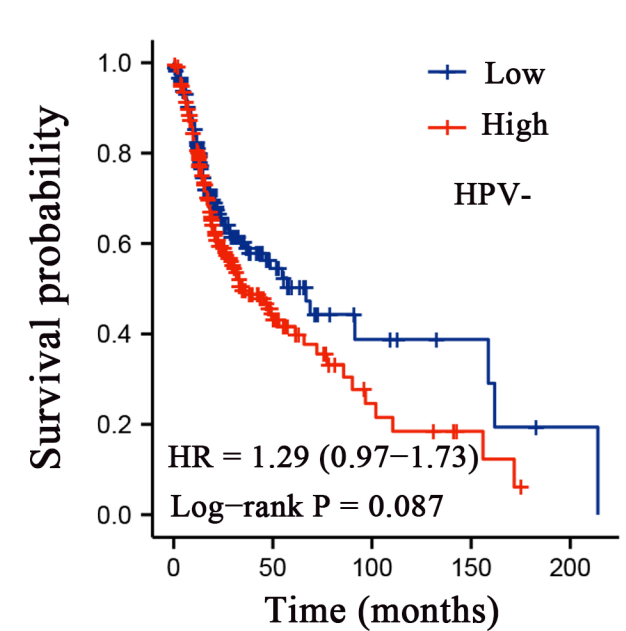

Supplement: Supplementary file 1 — Figure S1 [file CAM4-12-5703-s001.pdf]

A

TU177

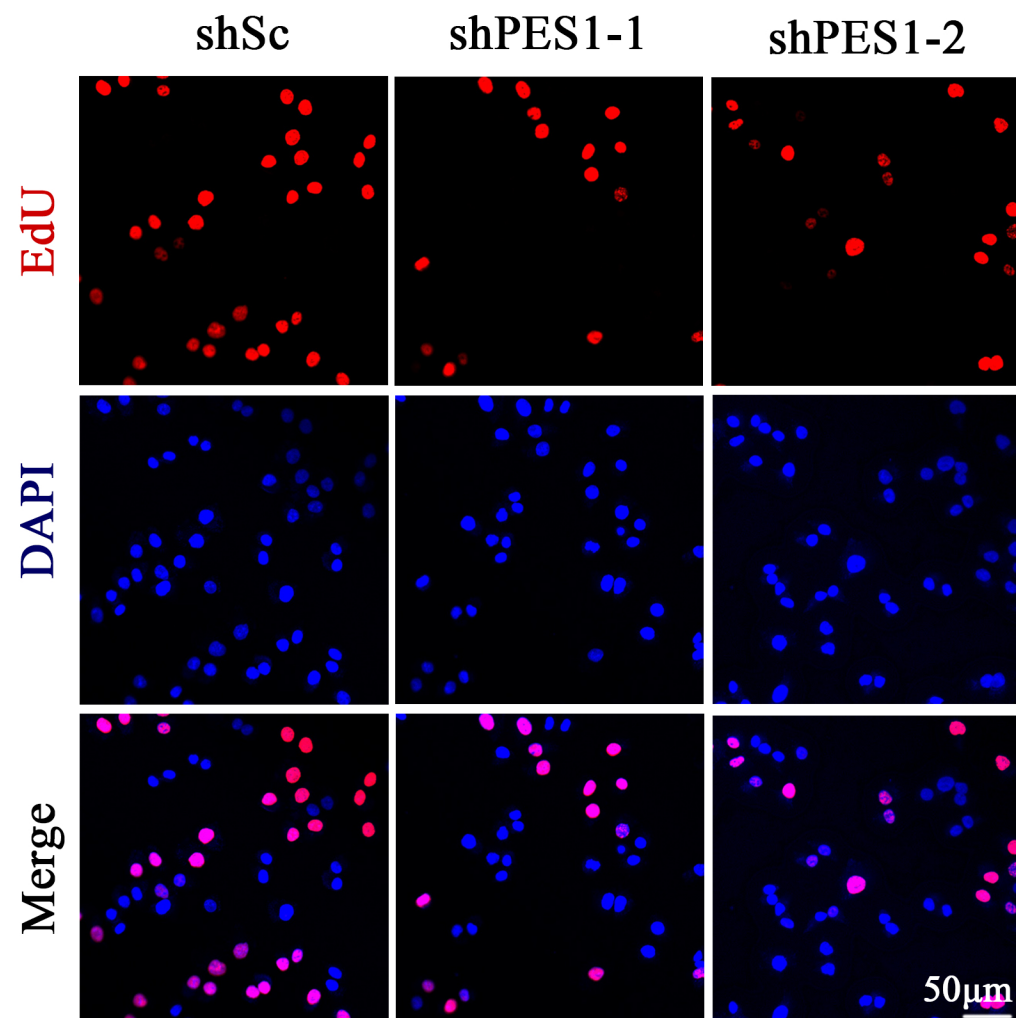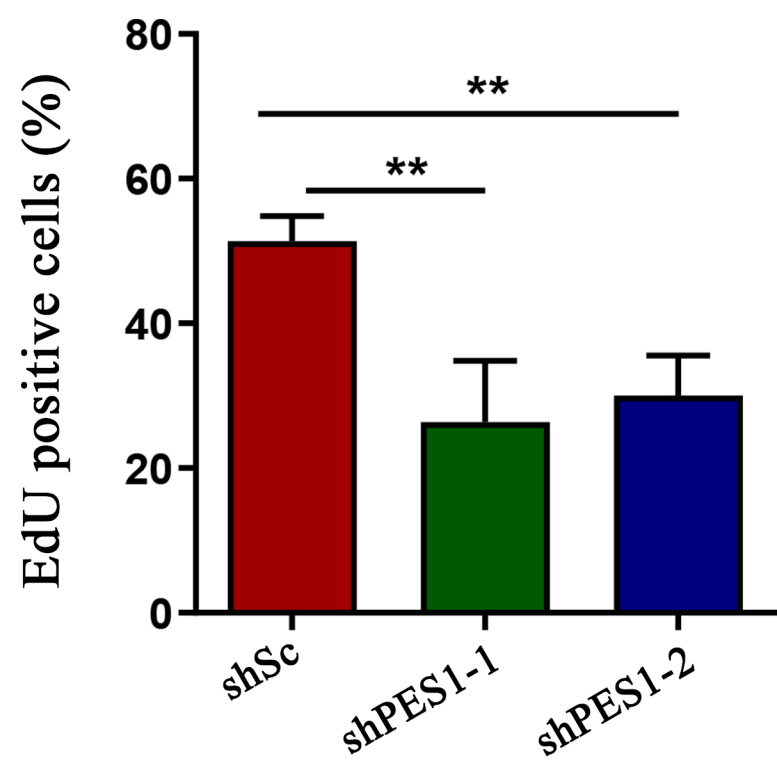

B

FaDu

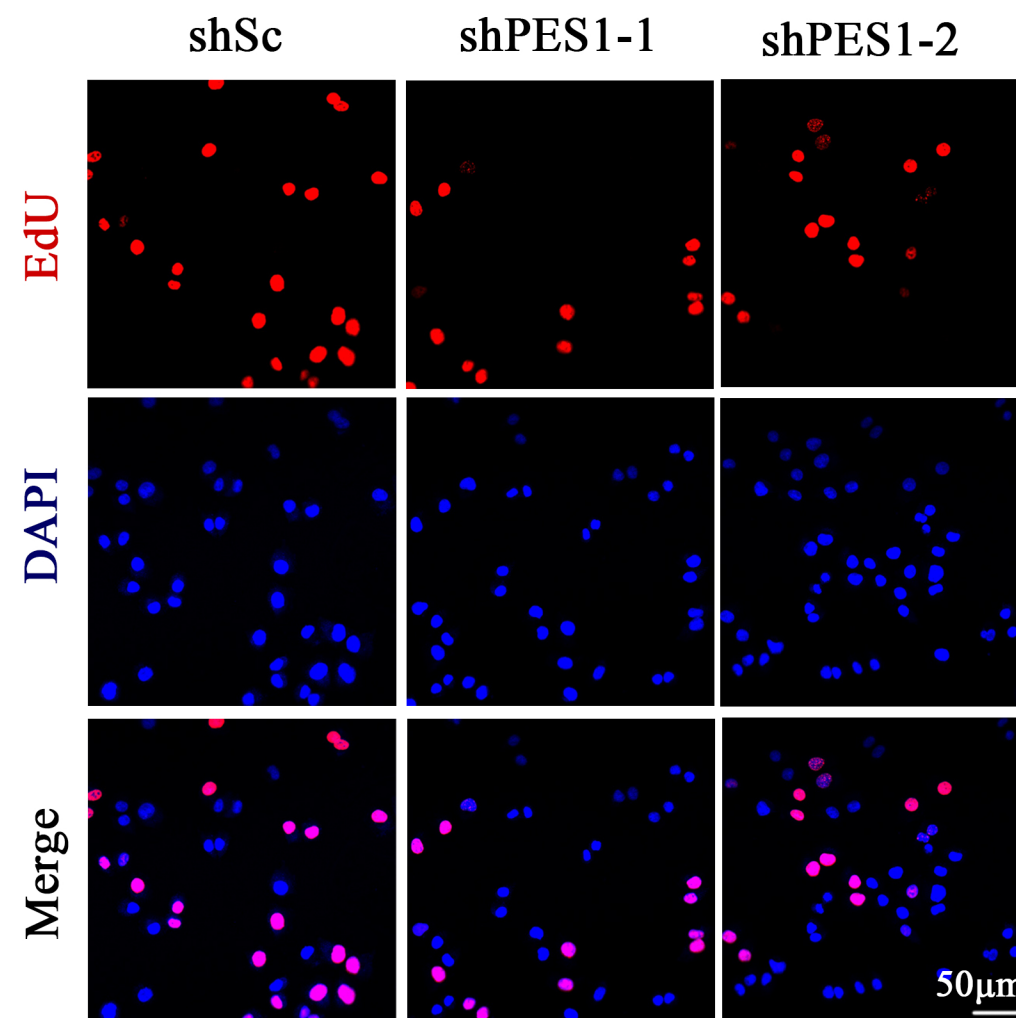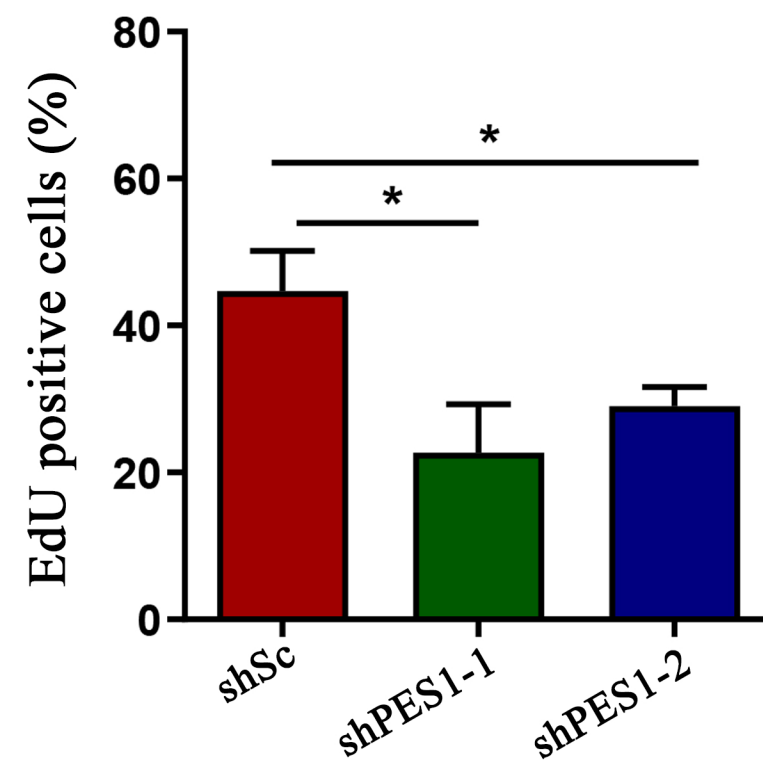

Supplement: Supplementary file 2 — Figure S2 [file CAM4-12-5703-s002.pdf]
